# Family History of Sudden Cardiac Death of the Young: Prevalence and Associated Factors

**DOI:** 10.3390/healthcare3041086

**Published:** 2015-11-09

**Authors:** Michelle J. White, Debra Duquette, Janice Bach, Ann P. Rafferty, Chris Fussman, Ruta Sharangpani, Mark W. Russell

**Affiliations:** 1 Department of Pediatrics and Communicable Diseases, Division of Pediatric Cardiology, University of Michigan, Ann Arbor, MI 48109, USA; E-Mails: mjoettewhite@gmail.com (M.W.); mruss@med.umich.edu (M.R.); 2 Genomics and Genetic Disorders Section, Michigan Department of Health and Human Services, Lansing, MI 48913, USA; E-Mails: DuquetteD@michigan.gov (D.D.); BachJ@michigan.gov (J.B.); rsharangpani@waynecounty.com (R.S.); 3 Chronic Disease Epidemiology Section, Michigan Department of Health and Human Services, Lansing, MI 48913, USA; E-Mails: annprafferty@gmail.com (A.P.R.); FussmanC@michigan.gov (C.F.)

**Keywords:** sudden cardiac death, genomics, family history, health disparities

## Abstract

Sudden cardiac death of the young (SCDY) is a devastating event for families and communities. Family history is a significant risk factor for this potentially preventable cause of death, however a complete and detailed family history is not commonly obtained during routine health maintenance visits. To estimate the proportion of adults with a family history of SCDY, the Michigan Department of Health and Human Services (MDHHS) Genomics Program included two questions within the 2007 Michigan Behavioral Risk Factor Survey (MiBRFS). Prevalence estimates and 95% confidence intervals were calculated. Among adults in Michigan, 6.3% reported a family history of SCDY, with a greater prevalence among blacks, those with lower household income, and those with less education. Among those reporting a family history of SCDY, 42.3% had at least one first-degree relative and 26.2% had multiple affected family members. This is the first study to demonstrate the prevalence of family history of SCDY while also highlighting key sociodemographic characteristics associated with increased prevalence. These findings should guide evidence-based interventions to reach those at greatest risk.

## 1. Introduction

Sudden Cardiac Death of the Young (SCDY) is a significant public health problem [1]. Each SCDY event is devastating to families and communities. These cases, often involving an ostensibly healthy individual, receive high profile media attention and prompt questions about prevention. In clinical studies, SCDY is usually defined as death prior to 40 years of age from an identified or suspected cardiac cause. However sudden cardiac death (SCD) has been variably defined in epidemiologic studies depending on available data [2].

Estimates of the incidence of SCDY in the United States vary widely from 0.5 to 20 per 100,000 person-years [1,3]. In Michigan the age-adjusted SCDY incidence is 5.5 per 100,000 [4]. Common causes of SCDY include heritable cardiomyopathies, primary electrophysiologic disorders, congenital heart disease and early coronary artery disease [3,5]. In many cases, the etiology remains unknown. There is an increased risk of SCDY associated with competitive athletic activity, which has led to an emphasis on screening children and adolescents prior to participation in competitive sports using a family history, a personal medical history, and a physical exam [6,7].

Family history is a significant risk factor for SCDY and represents an opportunity for prevention through directed screening/evaluation [8]. An estimated 60%–75% of causes for SCD are potentially heritable [9]. Additionally, many heritable diseases remain unidentified within families. A thorough family history paired with examination of relatives if indicated may lead to a more precise diagnosis and the identification of multiple individuals at risk [10]. Therefore, collection of family history is a vital first step to identify and manage individuals at risk for SCDY.

Multiple national professional medical societies have recognized the importance of family history in screening for individuals at risk of SCDY [7,10]. The American Heart Association recommends specific family history questions regarding SCDY in pre-participation athletic screening guidelines [11]. Despite this recommendation, the documentation of a detailed family history is often impeded by physician time constraints and patient recall [12,13]. To bridge this gap between recommendation and practice, the U.S. Surgeon General has promoted the importance of collecting family health history for clinicians as well as the public [14]. Two-thirds of U.S. and Michigan adults report that family history is important, however only one-third have actually collected their own family health history [15,16].

For over a decade in Michigan, SCDY has been recognized as a significant public health genomics issue. Epidemiological studies conducted by the Michigan Department of Health and Human Services (MDHHS) based on vital records data have shown an average of 326 out-of-hospital sudden deaths annually for Michigan residents between 1 and 39 years of age from 1999 to 2006 with higher rates in males, blacks and persons aged 35–39 [4].

MDHHS designed the current study to estimate the proportion of Michigan adults who have a family history of SCDY and to investigate its associations with demographic and health-related characteristics using data from the Michigan Behavioral Risk Factor Surveillance System (MiBRFSS).

## 2. Experimental Section

### 2.1. Survey Design

Michigan participates in the national Behavioral Risk Factor Surveillance System (BRFSS), which is coordinated by the Centers for Disease Control and Prevention (CDC) and is comprised of an annual, state-level telephone surveys of adults [17]. The 2007 Michigan Behavioral Risk Factor Survey (MiBRFS) followed the CDC BRFSS protocol and was conducted across the calendar year among a representative statewide sample of adults aged 18 years and older, selected through a random digit-dial sampling of landline telephone numbers followed by random intra-household selection of one eligible adult. The human research protection office at the MDHHS has determined that the annual surveys for the MiBRFS are exempt from institutional review.

The following questions, along with interviewer instructions, were developed, pre-tested, and included in the 2007 MiBRFS:

***Question #1:***  How many of your biological family members have had a sudden cardiac death, or sudden unexplained death, between the ages of 1 and 39?
***Question #2:***  What was their relationship to you?

Prior to the first question, the interviewer defined sudden cardiac death of the young as occurring when “a young, apparently healthy person dies suddenly from a cardiac arrest or from an unknown cause.” Interviewers were provided with instructions that the response should not include spouses or infants less than one year, drug-related deaths, traumatic deaths (such as car crashes), suicides, homicides, or individuals who had a long illness (cardiac or otherwise). Infants less than one year of age were not included due to the possibility of sudden unexplained infant deaths that could have numerous etiologies unrelated to SCDY. When responses to Question #2 included multiple family members with different relationships to the respondent, the interviewer was instructed to record the response verbatim.

Family history of SCDY was defined as having one or more biological family members who had experienced a sudden cardiac or unexplained death, between 1 and 39 years of age. Demographic information was gathered per routine including age, sex, race, education, and household income. The following variables from the core BRFSS were used to describe health-related characteristics: (i) currently has health insurance (yes/no); (ii) currently has a personal physician (yes/no); (iii) has had a routine checkup in the past year (yes/no); (iv) has had a blood cholesterol test in the past five years (yes/no); (v) rarely or never receives needed social and emotional support (yes/no); (vi) obesity (body mass index ≥ 30) (yes/no); and (vii) ever told by a doctor or other health professional that they had high blood pressure, high blood cholesterol (among those ever tested), diabetes, or cardiovascular disease (*i.e.*, heart attack, angina or coronary heart disease, or stroke) (yes/no). General health status was measured with the question, *“Would you say that in general your health is excellent*, *very good*, *good*, *fair*, *or poor?”* Disability was defined as a positive response to either of the following questions: *“Are you limited in any way in any activities because of physical*, *mental*, *or emotional problems?”* or *“Do you now have any health problem that requires you to use special equipment*, *such as a cane*, *a wheelchair*, *a special bed*, *or a special telephone?”*. No leisure-time physical activity was defined as a negative response to the question, *“During the past month*, *other than your regular job*, *did you participate in any physical activities or exercises such as running*, *calisthenics*, *golf*, *gardening*, *or walking for exercise?”*

The CDC BRFSS moderate and vigorous physical activity questions, which exclude occupational physical activity, were used to classify respondents’ degree of leisure-time physical activity. Adequate physical activity was defined as ≥30 minutes of moderate physical activity ≥5 days/week or ≥20 minutes of vigorous physical activity ≥3 days/week. Fruit and vegetable consumption was based on summed responses to the six core CDC BRFSS fruit and vegetable questions; inadequate consumption was defined as less than five times/day. Medicaid status was determined with the state-added question: *“Do you ersonally have Medicaid insurance?”* All data were self-reported.

### 2.2. Statistical Analysis

All statistical analyses were performed using SAS-callable SUDAAN statistical software to account for the complex survey design [18]. Data were weighted to account for the probability of selection and post-stratified to the Michigan adult population by age, race, and sex. Family history prevalence estimates and 95% confidence intervals (CI) were calculated by demographic characteristics, *i.e.*, age, sex, race, education, and household income. Prevalence and 95%CI of selected health-related characteristics were calculated by family history of SCDY status. Chi-square tests were used to assess overall bivariate associations. Multivariable logistic regressions were generated to test for significant differences in health-related characteristics by family history of SCDY after adjusting for age, sex, race, education, and household income.

## 3. Results

The BRFSS Council of American Survey Research Organizations (CASRO) response rate for the 2007 MiBRFS was 53.1%; the range among all participating states was 26.9% to 65.4%. The total working sample size for this analysis was 2856 individuals aged 18 years and above.

Overall, the proportion of adults in Michigan with a family history of SCDY was estimated to be 6.3% (Table 1). Nearly 5% of respondents (4.7%, 95%CI = 3.8–5.8) reported having one biological family member who had experienced a sudden cardiac or unexplained death between the ages of 1 and 39. 1.2% (95%CI = 0.7–2.1) reported having two family members with SCDY, while less than 1% had three or more family members who had experienced SCDY.

There was considerable variation by demographic groups. The proportion with a family history of SCDY was higher among blacks compared with whites (11.1% *vs*. 5.2%), higher among those with a high school education or less compared with those with at least some post-secondary education (9.3% *vs*. 4.5%), and higher among those living in households with incomes less than $50,000.

On multivariate analysis, the adjusted odds of having a family history of SCDY were significantly higher for females *vs*. males, black individuals *vs*. white individuals, individuals with less than or equal to a high school degree *vs*. those with higher education and for those with incomes of $20,000 to $49,999 compared to those with incomes greater than or equal to $50,000 (Table 2).

Among adults reporting a family history of SCDY (n = 190), nearly half (42.3%) reported at least one affected first degree family member (Table 3). Approximately one quarter of individuals with a family history of SCDY (26.2%) reported multiple affected family members. Among those with only one affected family member the most commonly reported affected family member was a sibling (brother or sister) at 17.4%, followed by cousin or a more distant relative at 16.5%.

**Table 1:** Estimated proportion of Michigan adults who have a family history of sudden cardiac death of the young ^*^ by demographic characteristics, 2007 Michigan Behavioral Risk Factor Survey [19].

Demographic Characteristics	n ^†^	% (95%CI)	*χ^2^ p*-Value
Total	2,856	6.3 (5.2–7.7)	
Age			
18–34	356	6.5 (4.1–10.3)	0.7550
35–64	1,677	6.0 (4.7–7.6)	
≥65	800	7.0 (5.0–9.7)	
Sex			
Male	1,006	5.4 (3.9–7.4)	0.0708
Female	1,850	7.7 (6.1–9.6)	
Race			
White	2,275	5.2 (4.1–6.6)	0.0123
Black	439	11.1 (7.7–15.9)	
Other	116	10.5 (4.7–21.6)	
Education			
≤High school graduate	1,104	9.3 (7.1–12.0)	0.0008
>High school graduate	1,744	4.5 (3.4–6.1)	
Household income			
<$20,000	413	7.8 (5.1–11.7)	0.0004
$20,000–49,999	975	8.6 (6.4–11.3)	
≥$50,000	1,079	3.5 (2.3–5.2)	

^*^ reported having at least one biological family member that had a sudden cardiac death, or sudden unexplained death, between the ages of 1 and 39; ^†^ Subpopulation cell sample size.

**Table 2:** Adjusted odds ^†^ of having a family history of sudden cardiac death of the young ^*^ among Michigan adults by demographic characteristics, 2007 Michigan Behavioral Risk Factor Survey [19].

Demographic Characteristics	Wald-F *p*-Value	Odds Ratio	95%CI
Intercept	0.0004	0.02	0.01–0.04
Age			
18–34	0.6128	0.98	0.49–1.94
35–64		1.24	0.73–2.11
≥65		(Ref)	(Ref)
Sex			
Male	0.0390	(Ref)	(Ref)
Female		1.57	1.02–2.42
Race			
White	0.0206	(Ref)	(Ref)
Black		1.86	1.15–3.01
Other		2.04	0.72–5.73
Education			
≤High school graduate	0.0233	1.72	1.08–2.75
>High school graduate		(Ref)	(Ref)
Household income			
<$20,000	0.0264	1.70	0.89–3.23
$20,000–49,999		2.18	1.24–3.84
≥$50,000		(Ref)	(Ref)

^†^ generated from a multivariable logistic regression with family history of SCDY as the dependent variable, and age group, sex, race, education, and household income as the independent variables; ^*^ reported having at least one biological family member that had a sudden cardiac death, or sudden unexplained death, between the ages of 1 and 39. Ref refers to the reference category used for that variable within the logistic regression model.

**Table 3:** Percent distribution of relationship of respondent to family member who experienced sudden cardiac death of the young (n = 190), 2007 Michigan Behavioral Risk Factor Survey [19].

Relationship to Respondent	% (95%CI)
≥1 first degree relative	42.3 (33.2–51.9)
*Multiple family members*	
≥1 first degree relatives	6.8 (3.8–11.8)
no first degree relatives	19.4 (11.6–30.6)
*Single family member*	
Parent	10.4 (6.2–16.9)
Sibling	17.4 (11.3–25.7)
Offspring	7.7 (4.4–13.0)
Aunt or uncle	6.4 (2.6–14.9)
Niece or nephew	9.5 (5.6–15.6)
Grandparent	1.1 (0.2–7.6)
Grandchild	0.6 (0.2–1.9)
Great grandparent, aunt, or uncle	4.4 (1.3–13.2)
Cousin or more distant	16.5 (10.2–25.5)

The prevalence of selected health care, health status, chronic conditions, and behaviors among those with and without a family history of SCDY are presented in Table 4. Among those with a family history of SCDY, a significantly higher proportion (23.1%) had Medicaid insurance compared with 10.6% among those without a family history (*χ^2^ p*-value = 0.0034). Additionally, those with a family history of SCDY had a significantly higher proportion of individuals who had been diagnosed with high blood pressure (39.5% *vs*. 27.9%) and a higher proportion who were current smokers (32.2% *vs*. 20.1%) compared to those without a family history of SCDY (*χ^2^ p*-values = 0.0131 and 0.0243 respectively). However, when tested within a multivariable logistic regression framework, high blood pressure was the only characteristic that remained significantly higher among those with a family history compared with those without, after adjusting for demographic variables.

**Table 4:** Prevalence of health-related characteristics among Michigan adults by family history of sudden cardiac death of the young *.

Health-Related Characteristic	Has Family History of SCDY ^*^		*χ^2^ p*-Value	Wald-F *p*-Value ^†^
	Yes % (95%CI)	No % (95%CI)		
*Health Care*				
No health insurance	17.8 (11.0–27.5)	10.6 (9.0–12.5)	0.1048	0.5798
On Medicaid insurance	23.1 (15.4–33.1)	10.6 (9.0–12.4)	0.0034 ^‡^	0.1971
No personal doctor	13.0 (7.9–20.5)	15.0 (12.9–17.4)	0.5396	0.5940
No routine checkup in past year	29.3 (20.1–40.5)	31.3 (28.7–34.0)	0.7059	0.9948
No blood cholesterol test in past 5 years	27.4 (17.9–39.6)	20.1 (17.6–23.0)	0.2286	0.3939
*Health Status*				
Fair to poor general health	16.1 (11.1–22.7)	14.3 (12.6–16.2)	0.5613	0.8687
Rarely-never receive needed emotional support	12.3 (7.1–20.4)	6.2 (5.1–7.6)	0.0773	0.0619
Has a disability	26.4 (19.6–34.6)	21.8 (19.8–23.9)	0.2296	0.1432
Obese (BMI ≥ 30)	34.0 (25.0–44.4)	27.6 (25.2–30.2)	0.2203	0.3252
*Chronic Conditions*				
Ever diagnosed with high blood pressure	39.5 (30.8–49.1)	27.9 (25.8–30.2)	0.0131 ^‡^	0.0019 ^‡^
Ever diagnosed with high cholesterol (among tested)	42.4 (33.0–52.4)	40.8 (38.1–43.5)	0.7492	0.7620
Ever diagnosed with diabetes	13.1 (8.9–19.1)	8.6 (7.5–9.9)	0.0801	0.0684
Ever diagnosed with cardiovascular disease	10.0 (6.2–15.8)	9.5 (8.3–10.9)	0.8345	0.9661
*Behaviors*				
Current smoking	32.2 (23.3–42.6)	20.1 (17.9–22.6)	0.0243 ^‡^	0.2078
No leisure-time physical activity	20.2 (13.3–29.5)	19.3 (17.2–21.5)	0.8199	0.8181
Inadequate physical activity	48.3 (38.2–58.5)	47.3 (44.5–50.0)	0.8542	0.8022
Inadequate fruit and vegetable consumption	82.4 (75.6–87.6)	78.1 (75.7–80.2)	0.1973	0.2502

^*^ reported having at least one biological family member who had a sudden cardiac death, or sudden unexplained death, between the ages of 1 and 39; ^†^ generated from multivariable logistic regressions with each health-related characteristic as the dependent variable, family history of SCDY as the independent variable, and age group, sex, race, education, and household income as possible confounding variables; ^‡^ *p* < 0.05.

## 4. Discussion

We have shown that a significant portion of the population endorses a family history of SCDY. To our knowledge, this is the first study to estimate the prevalence of family history of SCDY. The importance of family history has already been recognized in multifactorial cardiovascular diseases such as myocardial infarction and coronary artery disease. [20,21] As family history is a risk factor for SCDY, a complete family history may identify individuals at higher risk of SCDY. A complete family history should document the occurrence of known familial conditions associated with SCDY, and individuals who experienced sudden cardiac death or death of unknown causes. For affected family members the age of onset of symptoms, age at time of death and relation to the patient may also be important to guide further care [22]. Although a pre-participation screening for young athletes is often the trigger for such questions, family history, including family history of sudden cardiac death, should be a regular part of well-visits to the pediatrician/family care physician for children and young adults [23].

The health disparities found in our data are striking. The Michigan adult population is similar to that of the United States and therefore the socio-demographic composition of our survey population is potentially representative of the nation as a whole [24]. In addition to demonstrating a higher incidence of SCDY, blacks are also more likely to report a family history of SCDY than whites. Along with this racial disparity, individuals with a lower level of education and lower household income were more likely to report a family history of SCDY than those with more education and more income. To our knowledge these sociodemographic disparities have not been previously recognized. The National Heart Lung and Blood Institute Family Heart Study and Health Family Tree Study showed an inverse association between family risk score for coronary artery disease, stroke and hypertension and educational status in adults. There was also a higher incidence of these diseases in adults of lower educational status compared to adults of higher educational status [25]. Our findings may indicate a tendency toward earlier consequences of chronic cardiovascular disease in certain subpopulations. Further study is required, including evaluation of the etiologies and age at time of death for different sociodemographic groups, to determine the roots of these disparities.

The primary limitation of this study is that the family history of SCDY was self-reported, and not verified by medical records, death certificates, or autopsy reports. A second related limitation is that our data collection was susceptible to recollection bias. Notably, our data show the most common relative to be reported as a victim of SCDY was a sibling. Given the tragic and unexpected nature of SCDY, a sibling would likely be an accurate and reliable source of such information. Third, the MiBRFS is vulnerable to the limitations of all telephone surveys, including the potential bias from non-response and non-coverage.

Noting the significant health disparities in SCDY and the potential for screening and treatment of at-risk families, the following actions have been taken to reduce SCDY in Michigan:

□ Development, implementation and dissemination of formal recommendations for high school pre-participation screening forms, including personal medical history, family history, and physical examination based on the most current recommendations in the United States [11,26].
□ Education of health care providers and the public to increase awareness of SCDY and SCDY risk factors.
□ Promotion of the importance of documentation of a detailed family history of SCD for clinicians and the public.
□ Promotion of public awareness of cardiac symptoms and training in the use of cardiopulmonary resuscitation (CPR) and automated external defibrillators (AED).
□ Dissemination of emergency response protocols for victims experiencing sudden cardiac arrest.
□ Development of medical examiner protocols as a prevention opportunity for surviving family members.

## 5. Conclusion

Our study revealed a significant portion of individuals with family history of SCDY. Importantly, African Americans, lower income individuals, and individuals with less education were more likely to report a family history of SCDY. These findings should prompt further evaluation of the role of sociodemographic factors in SCDY. Furthermore, our findings underscore the importance of a detailed family history to identify those at greatest risk.
